# Remarks on Hepatic Affection, as a Cause of Other Diseases

**Published:** 1818-06

**Authors:** Archibald Robertson


Medico-Chirurgical

JOURNAL AND REVIEW.

VOL. V.] JUNE, 1818. [NO. 30.

### PART I.

### ORIGINAL COMMUNICATIONS.

—— quid utile ——

### *Remarks on Hepatic Affection, as a Cause of other Diseases.* By ARCHIBALD ROBERTSON, M. D.

THE existence of serious structural derangement of the liver, in circumstances where it is little or not at all suspected, and the influence which this derangement often has in sometimes causing, and at other times simulating, diseases of a very different character, have of late years been pressed, in a very forcible manner, upon my attention by the occurrence of many actual cases of that nature, and by the unequivocal appearances they presented on dissection. It therefore strikes me, that it may not be wholly useless to throw out a few hints on the subject, which, though confessedly imperfect and immature, may perhaps serve to draw the attention of the profession more strongly to this important matter.

In doing so, I am aware that I shall expose myself to the taunt of belonging to "The Hepatic School," and of viewing disease through a favourite medium, that converts every thing into its own proper hue; but I deem it a sufficient reply to say, that I am conscious of no hypothetical bias—for the opinions here advanced are deduced from facts and dissections, and that I belong to no School or Sect in Physic, ancient or modern, save that which proposes to establish the principles of the practice of Medicine on the basis of Morbid Anatomy.

It will not be irrelevant to the subject of this paper, if, before going farther, I make some comments upon the review of my Inaugural Dissertation in the March Number of your Journal, and explain those points which met not with your approbation. In doing this I can say, without affectation, that, in my own opinion, the strictures passed were probably much better merited than the praises bestowed upon that humble production: yet I am as free to confess my opinion, that these strictures lose much of their force, on my own mind at least, from the following considerations which I am now to submit to your readers.

The first stricture applies to my adopting too implicitly, and taking, as it were, for granted, the opinion of Dr. Clutterbuck, with regard to the brain being exclusively the seat of fever. Secondly, I am blamed for having omitted to lay down the diagnosis between the Dysentery treated of in my Essay, and the real Hepatitis of warm climates: And, thirdly, it is alleged that, in the cure, I ought to have employed blood-letting to even a greater extent than I appear to have done.

In replying to the first objection, I by no means intend to enter into the question at large about the proximate cause of Fever, a question which has proved an unsolvable difficulty to men of the first genius in every age; nor do I mean to defend, with any degree of tenacity, the opinion I happen to hold, being too well convinced of the futility of speculation, and the numerous sources of fallacy in matters of this kind, which are probably destined to be for ever hid from our eyes. Yet I believe the majority of modern Pathologists, after a very ample induction from facts, are now agreed in referring the primary cause of Idiopathic Fever—or rather the cause of those morbid movements constituting the complex idea attached to the term Fever — to a morbid condition of the centre of the nervous system; that is, of the brain and spinal-*marrow*, (as it is improperly called.) The latter is proved by the researches of Le Gallois and Magendie to be the residence of the *moving power* in every instance of voluntary motion. Hence, when excruciating pain of the back, torpor of the limbs, and prostration of all the voluntary powers, together with pain of head, imperfect sensation, confusion of the sensorium, and suppressed or impaired nervous energy, are found to occur as the earliest and first indications of disease,—often long before any commotion of the heart and arteries has yet taken place,—surely it is warrantable to draw the conclusion that the immediate, essential, and indispensable cause of fever consists in a morbid state of those central nervous masses contained within the cranium and spinal canal.

What this morbid state is we know not, and probably shall never know; but we are sufficiently sure of the fact: and from attending to the phænomena, we observe what, indeed, might *a priori* be expected from the close relation betwixt the nervous and vascular systems, that the latter sooner or later re-acts, as the phrase is, and thus fills up the full length portrait of fever. But this vascular re-action is only consecutive upon the original nervous derangement; and I am strongly inclined to believe, that the traces of early congestion and ultimate inflammation, observed in some of the thoracic or abdominal viscera, which have led Dr. Beddoes and others to refer the chief seat of Fever to one or other of these cavities, are also only consecutive, and the result of inequilibrium in the circulation of the blood—first thrown off its healthy balance by the antecedent disordered condition of the nervous system.

It would be interesting, no doubt, in a speculative point of view, to discover what this morbid condition is, just as it would be interesting to know what are the occult agencies that proximately cause the phænomena of attraction, of chemical affinity, or the like. But, for practical purposes, it is of little importance to penetrate these mysteries of Nature: for, as in the case of attraction, what we have to do is, not to discover the latent energy which causes it, but merely to study its laws; so, in the case of fever, all that we have to do is to note its phænomena and its laws, which is enough, in actual practice, to guide us to a right mode of cure. All that we have hitherto been able to observe of these phænomena and these laws; in short, the appearances observed as well during life as after death, impresses upon me the conviction that greater credibility is due to the opinion which considers the brain and spinal marrow as the seat of Fever, than to any other speculation heretofore advanced on this very dark point of Pathology. And this is the reason why I am 'a convert' to the views so well illustrated by Dr. Clutterbuck and other Pathologists: views which have been confirmed by dissections performed by myself* as well as others.

* For a detailed account of the morbid appearances of the brain and medulla oblongata in these dissections, I may be permitted to refer the

With regard to my having omitted to distinguish betwixt real Hepatitis and the disease I was treating of in my Essay, I shall only say, that I did so from conceiving the diagnosis to be self-evident. Hepatitis, I believe, is not attended with tormina, tenesmus, and numerous vitiated bloody stools, such as are observed in Dysentery.—Why the inflammation of the liver, coexisting with, and apparently causing, Dysentery in the New Orleans Epidemic, should excite and increase the action of the bowels, while in acute and chronic Hepatitis the alvine discharges are generally much diminished or suppressed, is a problem which I cannot otherwise solve than by supposing—and it is a supposition countenanced by the analogy of what takes place in the inflammations of other glands—that in the former case the hepatic inflammation was only to that degree as to modify and vitiate the bilious secretion (which consequently proved an acrid morbid stimulus to the intestines); whereas, in the latter case, (ordinary Hepatitis) the inflammation is exalted to such a degree as to suspend altogether the secretory function of this immense gland.

As to the third Stricture, namely, the alleged insufficient employment of the lancet in the methodus medendi, I own it is an imputation likely to be made by any one who considers merely the appearances on dissection, without knowing the fact I am now going to state, and which, perhaps, I ought to have stated in the body of the Dissertation, viz. that the majority of the fatal cases were men advanced in years, and extenuated by long servitude in tropical climates, or by former diseases; and who, by consequence, could not possibly bear extensive detraction of blood. Yet I will so far agree with my ingenious Reviewer, and admit the justice of his enlightened criticism, as candidly to confess, that had I the same painful duties to perform over again, I would certainly act up to his opinion—which indeed is also my own—and, *coute qui coute*, employ the lancet *more* freely in the cure, from a conviction that nothing but extensive depletion could arrest the structural and suppuratory derangement of the liver, and afford the patient a chance for life.

I was greatly pleased (and this brings me home more strictly to the subject of the present paper) to observe that my Reviewer did not attempt to throw any doubt upon the fact, that disease of the Liver is a very frequent

---

Reader to the 27th page of my paper in the second edition of Dr. Johnson's work on the Influence of Tropical Climates.

concomitant of Dysentery in warm climates, or to dispute my opinion that it is *the primary cause* of the severer forms of that complaint. I say, I was pleased with his forbearance in this respect, because, since my observations on the Winter Epidemic of New Orleans were published in the Edinburgh Medical Journal, about two years ago, almost every one has expressed surprize at the co-existence of extensive hepatic disease in these cases of Dysentery without any preceding evident indication of local mischief; nay, many have not scrupled to deny the thing altogether, and to assert, in contradiction to my positive observations, that the hepatic affection is a very rare occurrence, and merely a contingent — not a necessary — concomitant of Dysentery in warm climates. But I think I am warranted by facts in maintaining the contrary, viz. that the co-existence is very frequent, if not uniform; and that the connection between the two diseases is no less strict than that of cause and effect. Difficulties about the modus operandi of the one in exciting the other may be started, and may not, possibly, be able to be solved; but still they are merely theoretical difficulties, and in no way invalidate the fact itself: nor do I see that they invalidate the opinion about causation founded on that fact.

I can, however, well excuse a degree of scepticism on this point, knowing that what happened to myself may equally happen to others, that many cases of Dysentery may be examined after death, without the concomitant disease of the liver being discovered; for who would dream of cutting minutely into that viscus, in a disease that, according to established opinions in the Schools, is held to bear no relation to it.

It was by accident I discovered the fact, and I shall relate it simply as it happened. A Naval Officer,* of whose power of intellect, manly virtues, and elegant accomplishments I shall always entertain a respectful remembrance, whose memory I shall ever affectionately cherish, and whose premature decease I must ever regret, as the loss of a sincere and valued friend, was the first on board his Majesty's ship Cydnus that fell a martyr to Dysentery off New Orleans. He happened to die at sea, and it became desirable to preserve his remains until we should reach some port where the customary sepulchral honours due to his military rank might be decorously paid. In order to effect this, it was obviously necessary to remove the intes-

* The late Frederick Langford, Esq. Captain, R. N.

tines. While doing so, I ascertained that the liver was much enlarged, and therefore thought that it also had better be removed. Having separated it from its lateral connections, I passed my hand up under the ribs, to detach it from the diaphragm. While making a slight pressure for the latter purpose, I was astonished to feel the points of my fingers pass through the parietes of a large abscess in the upper and central part of the right lobe, from which upwards of a quart of pus forthwith flowed. After the liver had been removed, and laid out for minute inspection, I found the abscess of such extent as to admit my whole hand, and so lined in its inner surface with a thick, fretted, and irregular exudation of coagulable lymph, that it resembled a familiar and homely object; that is, a large winter glove lined with coarse worsted! On accurate examination, a second abscess was found, lower down in the large lobe, containing a pint of pus.

This officer had never, at any period of the disease, felt any pain in his side;—from his general intelligence, and from the accurate descriptions he gave me daily of his minutest sensations, I am convinced he would have mentioned that pain, had it existed even to the extent of a " sensus molestiae. Besides, he was one of the last men in the world that one would have suspected of hepatic affection, being florid in complexion, and having previously enjoyed the best health all his life.

Instructed by this insidious and lamented case, I had my eye to the liver ever afterwards; but pain of side, or pain on pressure under the ribs, was by no means often felt, though dissection after death brought to light hepatic disorganization equally extensive as in the above case. In many the liver, to appearance, had the colour and size of health; and it was not till on cutting into its parenchymatous substance that the extensive abscesses were discovered.

These things are of such high importance in the pathology of Dysentery, and so much depends upon the degree of credit that may be attached to them, that I am sincerely glad in being *now* able to say, that they no longer rest upon my solitary testimony or isolated observation. Within these few weeks I have been favoured with an interesting letter from James Simpson, Esq. Surgeon, R. N.; in which he details to me the cases, and appearances on dissection, of several Dysenterics treated by him in the East Indies. At the time he made the observations, he could not be aware that similar ones had been made by myself in the western hemisphere; therefore his remarks must carry with them the force of independent judgment and unbiassed observation. The symptoms before, and the morbid changes after death, were substantially—nay, exactly—the same as I have detailed in my Inaugural Dissertation, and in the preceding page. Mr. Simpson, speaking from the facts he has so often witnessed, expresses his belief that "future experience will unfold to us, that liver disease is an inseparable attendant of Dysentery in warm climates."

In addition to this strong testimony, I have reason to know that the experience of another gentleman, A. MacDonnell, Esq. of the 19th regiment, essentially coincides in this point with Mr. Simpson's and my own.

But Dysentery is not the only disease which owes its origin to a depraved condition of the liver and its secretions: this assertion I trust to vindicate in the sequel.

*Dyspepsia.* No complaint is more frequent in one or other of its various forms, in this country, than dyspepsia; and I am sure there are none of the experienced part of the Profession who have not felt how obstinately it, in general, resists the anti-acids, carminatives, tonics, and trumpery, usually prescribed for its cure. No doubt the stomach — placed as it is at the head of organic life, and sympathizing more or less closely with every part of the living machine — is, more than almost any other part of the body, liable to suffer, directly or indirectly, in its functions or its structure; unquestionably, too, its functions may be depraved even where there is no organic disease, simply by impaired vitality of its muscular and villous coats, which, by not acting duly on the ingesta, or by secreting a feeble or vitiated gastric juice, allow the alimentary mass contained in the stomach to run into fermentations, one while of the ascescent, and another while of the putrescent sort. Yet, while I admit this, I nevertheless hold the opinion, that unless where Dyspepsia arises from a sedentary life, excess of study, or of the depressing passions, (in which cases it is only temporary, and easily removed by avoiding the causes) it is, almost in every instance, *not* an idiopathic disease, but purely symptomatic of organic affection of the liver. Practitioners would be convinced of this would they but believe that chronic hepatitis is a much more frequent complaint in this country than we have been heretofore taught to suppose; and would they but pay attention to the colour of the alvine and renal discharges — to the pasty feel of the skin, and the dingy-pale hepatic hue of the countenance* that generally attend Dyspepsia; and would they but scrutinize the state of the liver when opportunities of dissecting such subjects happen to occur. It was by his attention, long and carefully directed to the condition of the liver and its secretions, and by his judicious administration of repeated doses of mercurials in dyspeptic or anomalous chronic complaints, that the justly-distinguished Mr. Abernethy has been enabled, in his work on "The Constitutional Origin of local Diseases," to give to Medical and Surgical Pathology one of the most valuable contributions which modern times can boast.

A very few weeks ago, I witnessed the dissection of a fatal case of Enteritis in a female, who had for years previously, been subject to cardialgia, acid eructations, torpid bowels,—in short, to the most exquisite dyspepsia. In this person, the liver was very much enlarged, though there was no appearance whatever of organic disease of the stomach or contiguous viscera. In this woman's case, diseased liver, had never been suspected during life, for she had no pain of side; and as for the enlargement of this viscus, I may remark by the way, that *it* must be very enormous ere it can be distinctly ascertained through the parietes of the abdomen.

*Phthisis Pulmonalis.* It might almost be inferred from *a priori* reasoning, that when a constant irritation is kept up on the system by visceral obstruction of the liver, the irritation will be propagated to the heart and lungs; and that the latter, in particular, will often ultimately become affected, owing to *sympathy of contiguity*—(an expression, which, though it explains nothing, sufficiently serves to express a recognized fact in pathological physiology:)— accordingly I have met lately with three cases where organic disease of the liver pretty accurately simulated the leading symptoms of phthisis. I question not but that they would eventually have terminated in real phthisis. Two of them were under my own care from the beginning, and I felt experimentally, how difficult was the diagnosis.

---

* It is curious to observe the sympathetic indication which the vessels of the tip of the nose afford of the state of the blood-vessels in the substance of the liver. When the apex of the nose is of a purple colour, its superficial veins large and tortuous, and its sides bestudded with a white pustular eruption, I have found it a sign, hitherto infallible, of organic affection of the liver. I have not, at this moment, time or means to ascertain whether this little circumstance has been mentioned in writings ere now. No doubt, ere now, it has been often observed.

In the other case, the patient was from the country, and his disease had actually been treated as phthisis for several months. He recovered, nevertheless, from a state that had been considered desperate, and was restored to perfect health by the steady use of calomel persevered in, with occasional intermissions, for four months, and repeatedly pushed to the length of salivation. The other two also completely recovered under similar means, and in one of them (that occurred very recently,) I found sponging the region of the liver, and the whole abdomen with the nitro muriatic acid mixture was a very considerable adjuvant in the cure, *after* the due employment of calomel.

Since these cases occurred, a valuable paper in the 7th vol. of the Medico-Chirurgical Transactions was pointed out to me by a friend. In that paper (on Dyspeptic phthisis) I find that views, in some respects analagous to my own, are brought forward by an observer of nature of no mean note or sagacity, Dr. Wilson Philip. On this point I gladly avail myself of the sanction of so respectable an authority.

*Diabetes Mellitus.* To assert that Diabetes, a disease whose pathology is still involved in utter darkness, and of which the treatment is purely empirical, owes its primary origin to organic disease of the liver, would be to assert much more than can be proved. Yet we all know the unhealthy state of the digestive organs, and the dirty, squalid, dusky hue of the skin, which usually precede and accompany this disease throughout; besides, in several dissections which I have witnessed of this singular malady, I have always observed the liver indurated or otherwise altered in its structure. The relations of this disease therefore with hepatic affection, deserve to be well investigated.

In September last, I was consulted in a case of diabetes of three years standing. The patient was of the atrabilious temperament, and had for a considerable time prior to the attack, laboured under chronic hepatitis. It was somewhat remarkable in this gentleman's case, that the increased discharge of urine would sometimes cease, and the saccharine matter disappear from it, and real ascites would succeed. As soon as the abdomen had obtained a considerable size, the diabetic symptoms would return, and the dropsical ones disappear. This alternation of ascites with diabetes occurred twice; at last, the patient, greatly reduced in strength and emaciated, suddenly became feverish, without any evident cause, and died in twenty-four hours afterwards.

It is worthy of remark in this case, that even before death the liver was long distinctly felt, all round the margin of the false ribs, very much enlarged, and as hard as a brick.

I purposely omit saying any thing of icterus, bilious diarrhœa, cholera, &c. which all the profession agree in referring to impaired function or structure of the liver, and which, consequently, come not within the aim of this paper. Here, therefore, I close my observations, regretting that they have run to so much greater a length than I intended. Of their comparative trivialness, no one can be more sensible than myself; nevertheless, they may not be altogether devoid of interest or utility; for, if I may judge from self-experience, communications in periodical works are sometimes not so useful from the mere information that they convey, (which, in cases like the present is very little,) as from the improving train of original reflection in the mind of the reader, which a casual suggestion, or even an erroneous speculation, will now and then excite. Should the above remarks be productive of any such beneficial effect, I shall not consider myself to have written in vain.

I am, &c.

*April*, 1818. ARCHIBALD ROBERTSON.

